# Editorial: Autophagy in Mammalian Development and Differentiation

**DOI:** 10.3389/fcell.2021.722821

**Published:** 2021-07-27

**Authors:** Sabrina Di Bartolomeo, Lucia Latella, Konstantinos Zarbalis, Federica Di Sano

**Affiliations:** ^1^Department of Biosciences and Territory, University of Molise, Pesche, Italy; ^2^Institute of Translational Pharmacology, National Research Council of Italy, Rome, Italy; ^3^Epigenetics and Regenerative Medicine, IRCCS Fondazione Santa Lucia, Rome, Italy; ^4^Department of Pathology and Laboratory Medicine, University of California, Davis, Davis, CA, United States; ^5^Institute for Pediatric Regenerative Medicine, Shriners Hospitals for Children, Sacramento, CA, United States; ^6^MIND Institute, University of California, Davis, Davis, CA, United States; ^7^Department of Biology, University of Rome “Tor Vergata,”Rome, Italy

**Keywords:** autophagy, differentiation, stem cells, reprogramming, lysosomes

Autophagy is a cellular degradation and recycling process by which cytoplasmic components, including macromolecules and organelles, are sequestered into specialized autophagosomal vesicles to be delivered and degraded in lysosomes (Klionsky and Emr, 2000; Levine and Klionsky, 2004; He and Klionsky, 2009). Autophagy is highly active during the earliest stages of development regulating stem cell pluripotency and differentiation (Jang et al., 2016; Xu et al., 2020), but also fundamental to later morphogenetic processes that together with apoptosis are decisive in tissue remodeling and shaping the embryo's organization (Qu et al., 2007). Moreover, autophagic process likely protects cells during metabolic stress and nutrient deprivation that occur during tissue remodeling. It is therefore evident that the close interplay between autophagy and the processes of cell death, proliferation, and differentiation determine eukaryotic development.

This Research Topic aimed at providing further context to the role of autophagy in orchestrating cellular development, differentiation, and aging in both physiological and pathological conditions.

In this context Perrotta et al. reviewed recent findings that further illuminate autophagy's impact on differentiation and maintenance of endothelium, muscle, immune system, and brain. Their detailed description provides a comprehensive framework of emerging results and highlights the pivotal role of autophagic response in a multitude of tissue functions that critically depend on stem cell maintenance and differentiation.

Campanario et al. describe two strategies for assessing autophagic activity in satellite cells. Adult skeletal muscle has the capability to regenerate by virtue of its resident stem cells (satellite cells). With aging, satellite cell regenerative capacity declines, correlating with loss of autophagy. Enhancing autophagy in aged satellite cells restores their regenerative functions, underscoring this proteostatic activity's relevance for tissue regeneration. Thus, the methods presented in this study allow a rapid assessment of autophagic flux in muscle stem cells by flow cytometry and immunofluorescence enabling researchers in investigating the role of autophagy in muscle homeostasis, regeneration and diseases (Campanario et al.).

Reactivating autophagy in COL6 null mice ameliorates the myopathic phenotype. The findings by Metti et al. point at the effects of pterostilbene (Pt), a non-toxic polyphenol belonging to the stilbenoid family, on skeletal muscle homeostasis. The authors show that Pt is an effective autophagy-inducing nutraceutical for skeletal muscle with great potential in counteracting the major pathogenic hallmarks of COL6-related myopathies, a valuable feature that may be also beneficial in other muscle pathologies characterized by a defective regulation of the autophagic machinery.

These data point at Pt as an effective non-toxic, caloric restriction mimetic that can be exploited for the treatment of autophagy-deficient pathologies (Metti et al.).

Cui et al. found that miR-204 is highly expressed in chicken atrophic ovaries promoting granulosa cell apoptosis *via* repressing FOXK2 through PI3K/AKT/mTOR pathway and inhibited autophagy by impeding the TRPM3/AMPK/ULK pathway. In fact, it is known that ovarian tumor is accompanied by an increase in follicular atresia, cell apoptosis, and autophagy (Ma et al., 2019). Functions of miR-204 have been linked to many biological processes, including maintenance of joint homeostasis and protection against osteoarthritis, tumor growth, migration and anoikis of cancer cells, and autophagy. This study could be useful in identifying the regulators of miR-204 target genes and their roles in signaling pathways associated with both the development and function of the ovary.

Histone deacetylases (HDACs) are a group of enzymes that remove acetyl groups from both histone and non-histone proteins Zhan et al. found that HDAC6 regulates the fusion of autophagosome and lysosome during odontogenesis. Interestingly, this study provides a new viewpoint into the role of autophagy in odontoblast differentiation.

In the past few years, our understanding of the interplay between autophagy and genomic stability has greatly increased and several papers suggested a molecular connection between the DNA damage response (DDR) and autophagy.

Ataxia-telangiectasia mutated kinase (ATM) is the product of a gene whose mutation leads to the development of a rare genetic neurodegenerative disorder, Ataxia-telangiectasia (A-T). Importantly, ATM kinase plays a central role in the DDR and it can finely tune the balance between senescence and apoptosis: activated ATM promotes autophagy and sustains the lysosomal–mitochondrial axis. In the review by Stagni et al., recent advances in understanding the molecular mechanisms linking DNA damage, oxidative stress and autophagy to senescence are summarized, pointing out the role of ATM kinase in these cellular responses. The significance of this regulation in the pathogenesis of A-T is also discussed.

Still regarding the role of autophagy in detoxifying the cellular environment and ensuring cellular survival, we report the work from Vanasco et al.. The authors identify a novel DRP1-Parkin1-VMP1 selective autophagy pathway, which mediates the selective degradation of damaged mitochondria by mitophagy in acute pancreatitis therefore restoring mitochondrial functions (Vanasco et al.).

The role of Oleic Acid (OA) in hepatocellular carcinoma (HCC) through autophagy has been investigated by Giulitti et al.. OA is one of the most abundant monounsaturated fatty acid representing the main component of olive oil (70–80%) that has beneficial effects in counteracting liver steatosis and cardiovascular diseases (Perez-Martinez et al., 2011; Perdomo et al., 2015).

The authors report a OA- specific effect on lipid accumulation, viability, proliferation, migration, and invasion which is partially due to a reduced autophagy leading to an OA anti-tumor effect in HCC.

Lastly, it has been observed that autophagy inhibition through poly (ADP-ribose) polymerase-1 (PARP1) inactivation protects cardiomyocytes from Myocardial ischemia–reperfusion injury (MIRI), characterized by post-ischemic cardiomyocytes death and reperfusion myocardial damage (Xu et al.)

We hereby thank all the authors that participated in this Research Topic. Their articles significantly contribute to a more comprehensive understanding of the roles that autophagy plays in regulating different aspects of mammalian development and differentiation and add new concerns to uncovering mechanisms underlying human disease.

## Author Contributions

FD and SD conceived and wrote the Editorial. LL and KZ reviewed and edited the manuscript. All authors contributed to the article and approved the submitted version.

## Conflict of Interest

The authors declare that the research was conducted in the absence of any commercial or financial relationships that could be construed as a potential conflict of interest.

## Publisher's Note

All claims expressed in this article are solely those of the authors and do not necessarily represent those of their affiliated organizations, or those of the publisher, the editors and the reviewers. Any product that may be evaluated in this article, or claim that may be made by its manufacturer, is not guaranteed or endorsed by the publisher.
